# Balance as an Additional Effect of Strength and Flexibility Aquatic Training in Sedentary Lifestyle Elderly Women

**DOI:** 10.1155/2020/1895473

**Published:** 2020-01-25

**Authors:** Fernando Alves Vale, Mariana Callil Voos, Christine Brumini, Eneida Yuri Suda, Ronaldo Luis da Silva, Fátima Aparecida Caromano

**Affiliations:** ^1^PT, MSc. Rehabilitation Sciences Post Graduation Program of Medical School of University of São Paulo, Laboratory of Physical Therapy and Behavior, São Paulo, Brazil; ^2^Professor at Ibirapuera University, Physical Therapy Course, São Paulo, Brazil; ^3^PT, PhD. Rehabilitation Sciences Post Graduation Program of Medical School of University of São Paulo, Laboratory of Physical Therapy and Behavior, São Paulo, Brazil; ^4^PT, MSc. Rheumatology Sciences, Federal University of São Paulo, São Paulo, Brazil; ^5^PT, MSc. Faculté des Sciences, Université du Québec à Montréal, Quebec, Canada; ^6^PT, MSc. University Center São Camilo, São Paulo, Brazil

## Abstract

**Objective:**

To evaluate the additional effects of on balance an aquatic muscle strengthening and flexibility training program in healthy sedentary lifestyle elderly women.

**Method:**

This controlled clinical trial included 56 healthy sedentary women, aged from 65 to 70 years, divided into two groups. The aquatic group (AG) underwent aquatic training (45 minutes/session, 32 sessions), and the control group (CG) received no intervention. Data were collected pre- and post-intervention, during a one-week period. Lower limb muscle strength was measured by a force sensor (myometer). Flexibility was measured by biophotogrammetry. Functional balance was evaluated by the Performance Oriented Mobility Assessment (POMA) and the Berg Balance Scale (BBS).

**Results:**

Muscle strength, flexibility, and balance improved in AG (*p* < 0.001), but not in CG.

**Conclusion:**

The aquatic exercises program, which was originally developed to promote muscle strength and flexibility, also improved functional balance. Aquatic training is an option for physical health promotion for sedentary lifestyle elderly women.

## 1. Introduction

In a previous study, we observed that aquatic training improved muscle strength and flexibility in sedentary lifestyle elderly women [1, 2]. However, some participants also mentioned balance improvement after aquatic training. In the present study, we replicated the previous experimental protocol, to study possible functional balance benefits after the muscle strength and flexibility aquatic training program. Sedentary behavior is inversely associated with quality of life in older adults. Besides, it has been associated to a higher risk of developing obesity, type-2 diabetes, bone hypodensity, and cardiovascular diseases [1].

Aquatic exercises are well accepted by older adults for muscle strength training. Mobility is optimized by the physiological effects triggered by warm water immersion, associated with specific kinesiological challenges imposed by the aquatic environment, such as constant postural readjustment [3, 4]. Water supportive property facilitates antigravity muscles contraction. The hydrostatic pressure provides longer times for vestibular processing for postural reactions and better movement perception. Therefore, older adults with risk of falls can perform functional exercises safely [5–7].

Studies have shown that muscle strength exercises contribute to functional balance improvement in older adults [8–10]. Therefore, muscle strengthening should be considered not only to reduce falls, but also to reduce the fear of falling and depression [11]. Westhoff et al. [12] demonstrated the effectiveness of a muscle strength aquatic training program. Older adults improved the functional independence in daily life activities, showing that there was a correlation between muscle strength and functional independence. According to Rose and Clark [13], aquatic exercises can contribute to dynamic balance improvement and this objective must be considered for programs.

Aquatic training programs usually specify pool depth, movement velocity, and direction, verbal command and cues, and frequency and intensity of exercises [14, 15]. Studies have been carried out to evaluate the possibilities of different environments to promote muscle strength and balance improvement, especially considering the aquatic exercise programs for older adults [1, 2, 4, 16, 17].

Aquatic exercises have been proposed to treat elderlies with several diseases, such as knee osteoarthritis, stroke, and chronic low back pain. Besides muscle strength, and functional independence, the programs involve cardiorespiratory training [18–21], but few studies investigated balance improvement. The objective of this study was to investigate possible beneficial effects on functional balance after strength and flexibility aquatic training in sedentary lifestyle elderly women.

## 2. Method

### 2.1. Study Design

This controlled clinical trial evaluated 52 participants, randomly divided by simple draw, into two groups. The study was approved by the Hospital Ethics Committee of the Faculty of Medicine of the University of São Paulo (Protocol 2.327.178) and followed the Declaration of Helsinki [22].

### 2.2. Subjects

Seventy women, who declared themselves as healthy and as having a sedentary lifestyle, aged 65–70 years, were invited to participate in this study. Inclusion criteria were nonsmoking; not having practiced, in the last two years, physical exercises, nor walking over one kilometer more than twice a week.

Women who referred musculoskeletal, neuromotor or cardiovascular dysfunction, or chronic diseases that could be a risk during aquatic physical activities were excluded. The required time availability and mobility (not missing the sessions due to transport difficulties) and accepting the training routine were also explained prior to the study initialization. All absences were justified and missed sessions were replaced.

Fifty-eight women signed the Free and Informed Consent Form and were randomly divided into two groups: aquatic group (AG, *n* = 28) and control group (CG, *n* = 28). All participants were clinically assessed prior to starting the protocol. During the training period, four women were excluded and therefore 52 women completed the program (AG, *n* = 26 and CG, *n* = 26).

Two participants were excluded because they missed two classes in the same week, which could not be replaced, one woman declared lack of interest in the proposed physical activities, and three participants had clinical intercurrences (renal infection, dermatitis and a tibial fracture in a car accident).

The mean age of AG was 67.3 years (±1.7), the mean weight was 74.3 kg (±8.8) and the mean height was 162.3 cm (±3.3). The mean age of the CG was 67.3 years (±1.7), the mean weight was 74.2 kg (±11.8) and the mean height was 162.0 cm (±3.7). Both groups presented average body mass index classified as overweight (28.3 kg/m^2^). There were no statistical differences between groups characteristics.

### 2.3. Assessment

Muscle strength was evaluated by a force sensor (myometer, Lafayette Instrument ®). Isometric muscle strength of hip flexors, knee extensors, ankle extensors, and ankle flexors were performed on both sides. The subjects were verbally encouraged to produce the maximum possible force. The tests were repeated three times, and the best score was registered for each side and muscle group.

Flexibility was evaluated by standardized photographic measures of individuals using the simple third finger-to-floor distance test (modified to wrist-to-floor). The wrist-to-floor distance was measured during trunk flexion. Distances were measured directly on the printed photos, with a pachymeter and a square. The pachymeter is used to measure the wrist-to-floor distance between the ulna styloid process marked with a white sticker of 1 cm diameter [23] and the ground. The results of the third finger-to-floor test are usually zero, that is, the finger touches the floor. Considering that the hand is about 20 centimeters long, the results obtained in the present study were approximately 20 cm apart from the ground.

The Performance Oriented Mobility Assessment (POMA) was developed by Tinetti [24], with translation, cultural adaptation, and validation to Brazilian Portuguese [25]. The Brazilian version is composed of two evaluations related to balance and gait. The performance on 13 activities that replicate daily movements that require balance is scored from 13 to 39. In gait section, nine items are observed during continuous walk by a path and scored from 9 to 18. The results of the two domains are added to obtain the final score. Higher indices indicate better balance.

The Berg Balance Scale, developed by Berg et al. [26], was translated and adapted to Brazilian Portuguese by Miyamoto et al. [27]. It consists of 14 common daily tasks. The maximum score is 56 and each task ranges from 0 (unable to perform task) to 4 (done independently). Between 54 and 46 points, decreasing one point is associated with a 6%–8% increase in the risk of falls, and scores below 36 mean that the risk is almost 100%.

### 2.4. Intervention

All participants were assessed with muscle strength (myometer isometric test), flexibility (wrist-to-floor test), POMA, BBS and then randomly divided in AG or CG. The AG started the exercise program, which lasted for four months, twice a week, in one-hour sessions. The aquatic training was performed in a pool with dimensions of 6.0 × 4.0 m and 1.3 m depth at a mean temperature of 31.5°C (31.0°C–33.0°C). The Laboratory of Physical Therapy and Behavior of the Physical Therapy Course of the University of São Paulo Medical School was used for data analysis.

The AG performed the strength and flexibility training program, proposed by Candeloro and Caromano [2]. The aquatic training consisted of 32 sessions, during 16 consecutive weeks. The first four sessions (pre-training) were used to adapt to the aquatic environment, and remaining 28 sessions were used for flexibility and muscle strength training, with up to seven levels of increasing difficulty exercises. The sessions were performed in pairs and lasted for one hour, with 10 minutes for vital signs measurement and 50 minutes for motor activities training. The exercise program provided 29 motor activities: 6 were warm-up activities, 8 muscle strengthening exercises, 11 flexibility training activities, and 4 relaxation activities. The exercises (the use of floats for resistance and the number of repetitions) varied according to the degree of difficulty, determined by group performance.

For CG, there was no intervention. The participants only performed the physical-functional evaluations at the beginning of the study, and after four months. After that the end of the present study, the CG participants were invited to start the aquatic program (the same protocol offered to AG).

### 2.5. Statistical Analysis

Means and standard deviations for continuous variables and percentages for categorical variables described the sample and the variables. The Shapiro-Wilk test was used to verify data normal distribution. The analysis of intragroup data (before and after intervention) was performed by paired student *T* test, whenever there was normal distribution and Wilcoxon test was used for nonnormal distribution data.

The significance of the intergroup differences (AG vs. CG) was assessed using the nonparametric Mann-Whitney test and the parametric independent Student *T*-test. Correlation analyzes were performed using Pearson correlation test. All analyses were performed with SPSS Statistics 21.0 (IBM, Arkmon, NY). The study admitted the value of *p* < 0.05 for statistical significance.

## 3. Results

Table 1 presents the values of muscle strength and flexibility before and after intervention of each group and the comparisons between groups. The wrist-floor distance significantly decreased after intervention in the AG only (*p* < 0.001). The intergroup difference showed a greater flexibility gain in AG compared to CG. Similarly, muscle strength increased significantly in knee, hip, and ankle segments.

POMA and BBS scores showed statistically significant improvement after intervention, in AG.  Table 2 shows the values obtained before and after the intervention.

## 4. Discussion

The present study investigated possible beneficial effects on functional balance after strength and flexibility aquatic training in sedentary lifestyle elderly women. Although the training program focused on strength and flexibility, the AG also showed better functional balance scores (POMA and BBS) after intervention. Strength, flexibility, and balance may be affected by aging. Therefore, interventions with physical exercises for older adults may reverse strength, flexibility, and balance loss and reduce the risk of falls.

The AG presented improvement in muscle strength, flexibility, POMA, and BBS scores, while the CG did not. The CG remained with no intervention during the study period and was only offered the same protocol given to AG after the end of the present study. Additional balance improvement after aquatic strength and flexibility training corroborates the findings of Avelar et al. [9]. These authors showed that water or soil resistance programs improved the static and dynamic balance in elderly women, evaluated by the dynamic gait index, BBS, and tandem gait. The muscle strength improvement optimized the recruitment of ankle, knee, and hip muscles in postural strategies [16, 28, 29]. Strength improvement contributes to a lower number of stumbles and falls.

Simmons and Hansen [30] compared soil and aquatic exercise groups in older adults using the Functional Reach Test. An improvement was observed in the aquatic training group, after five weeks. Postural reactions can be planned and corrected safely in the aquatic environment. A greater joint range of motion is possible in water immersion, which also stimulates proprioception, also optimized by the constant feedback given by water resistance. BBS improvement was also observed by Resende and Rassi [17] after an aquatic program of low to moderate intensity. Participants performed stretching exercises, and static and dynamic balance training for 12 weeks.

Aquatic exercises interventions improved adherence to rehabilitation programs, such as SWEATTM method, described by Sanders et al. [10]. These authors proposed an effective shallow and deep-water training that improved cardiovascular and muscular endurance in women aged 60–89 years. The program lasted for 16 weeks and included warming-up, functional, cardiovascular, and strengthening exercises. The most relevant gains were in static balance and in sit-to-stand transferring. However, the walking speed, agility and flexibility also improved after training.

Meereis et al. [31] evaluated institutionalized older adults after aquatic strength and flexibility training. The authors observed that the BBS scores improved after training, which corroborates the results of Avelar et al. [9], Ballard et al. [32], Meereis et al. [31], Resende and Rassi [17], and the present study. Although the mean BBS and POMA scores of both groups of the present study were already relatively high prior to training, the AG showed a statistically significant improvement after the intervention. The benefits of aquatic training on postural control may be even higher in populations with more severe postural impairment.

Conventional (soil) programs improve balance and should be encouraged [32], because they provide different postural feedback, compared to aquatic training. Therefore, aquatic and soil training may be practice complementary skills. Although the resources needed for aquatic training may be challenging, the beneficial physical and psychosocial effects must be considered in clinical decision making [33–36]. The present study supports the idea that aquatic training is highly suitable for older adults and can be considered health promotion programs. Randomized controlled trials, with control groups and blind evaluators, are suggested for future studies, to investigate the effects of aquatic training to improve balance in older adults. Different programs of aquatic and soil exercises involving balance, strength, and flexibility training should also be compared.

## 5. Conclusion

Sedentary lifestyle elderly women benefitted from aquatic training and improved muscle strength (measured by the myometer isometric test), flexibility (measured by wrist-to-floor test) and functional balance (POMA and BBS scores), compared to a nontrained control group.

## Figures and Tables

**Table 1:** Muscle strength and flexibility before and after intervention.

Variable	AG			CG						Confidence interval		
	*N* = 26			*N* = 26								
	Pre-intervention	Post-intervention	Intragroup *p*	Pre-intervention	Post-intervention	Intragroup *p*		Intergroup *p*		Effect size	Lower limit	Upper limit
Hip flexors strength (kgf)	14.3 ± 2.4	18.8 ± 2.3	<0.001	14.4 ± 2.5	14.6 ± 2.9	0.409		<0.001		−1.61	−2.48	−0.72
Knee extensors strength (kgf)	14.2 ± 2.7	18.2 ± 2.7	<0.001	14.7 ± 2.8	14.8 ± 2.6	0.542		<0.001		−1.28	−2.12	−0.44
Ankle flexors strength (kgf)	11.3 ± 1.5	14.2 ± 1.6	<0.001	11.5 ± 1.6	11.6 ± 1.9	0.565		<0.001		−1.48	−2.35	−0.61
Ankle extensors strength (kgf)	11.2 ± 1.3	14.2 ± 1.6	<0.001	11.9 ± 1.3	11.3 ± 1.5	0.611		<0.001		−1.87	−2.79	−0.95
Wrist-floor distance (cm)	34.7 ± 131.8	14.8 ± 8.4	<0.001	35.4 ± 15.0	35.8 ± 14.9	0.069		<0.001		1.77	0.86	2.68

*N*: number of participants, mean ± standard deviation, intragroup *p*: statistical difference between pre-intervention and post-intervention periods in each group using Wilcoxon and *T* tests, intergroup *p*: statistical difference between groups using Mann;–Whitney and *T* tests, effect size used Cohen test. AG: aquatic group, CG: control group.

**Table 2:** Functional balance before and after intervention.

Variable	AG			CG				Confidence interval		
	*N* = 26			*N* = 26						
	Pre-intervention	Post-intervention	Difference	Pre-intervention	Post-intervention	Difference	Intergroup *p*	Effect size	Lower limit	Upper limit
POMA score	36.8 ± 1.1	38.1 ± 0.7	1.3 ± 0.8a	36.7 ± 0.9	36.6 ± 0.8	0.1 ± 0.6	<0.001	−1.99	−1.06	−2.94
BBS score	53.4 ± 1.8	54.9 ± 1.2	1.4 ± 1.0a	53.3 ± 1.2	53.1 ± 1.5	0.2 ± 0.5	<0.001	−1.33	−2.17	−0.48

*N*: number of participants, mean ± standard deviation, a: statistically significant difference between pre- and post-intervention periods. Effect size used Cohen test.

## Data Availability

Data is available if consulting is necessary.
